# EcoWild: Reinforcement Learning for Energy-Aware Wildfire Detection in Remote Environments

**DOI:** 10.3390/s25196011

**Published:** 2025-09-30

**Authors:** Nuriye Yildirim, Mingcong Cao, Minwoo Yun, Jaehyun Park, Umit Y. Ogras

**Affiliations:** 1 Department of Electrical and Computer Engineering, University of Wisconsin-Madison, Madison, WI 53706, USA; nyildirim@wisc.edu (N.Y.); mcao53@wisc.edu (M.C.); 2 Department of Electrical, Electronic and Computer Engineering, University of Ulsan, Ulsan 44610, Republic of Korea; yma1591@ulsan.ac.kr

**Keywords:** Wildfire detection, RL, energy-aware sensing, embedded systems, cyber-physical systems, solar-powered devices, edge computing, environmental monitoring

## Abstract

Early wildfire detection in remote areas remains a critical challenge due to limited connectivity, intermittent solar energy, and the need for autonomous, long-term operation. Existing systems often rely on fixed sensing schedules or cloud connectivity, making them impractical for energy-constrained deployments. We introduce EcoWild, a reinforcement learning-driven cyber-physical system for energy-adaptive wildfire detection on solar-powered edge devices. EcoWild combines a decision tree-based fire risk estimator, lightweight on-device smoke detection, and a reinforcement learning agent that dynamically adjusts sensing and communication strategies based on battery levels, solar input, and estimated fire risk. The system models realistic solar harvesting, battery dynamics, and communication costs to ensure sustainable operation on embedded platforms. We evaluate EcoWild using real-world solar, weather, and fire image datasets in a high-fidelity simulation environment. Results show that EcoWild consistently maintains responsiveness while avoiding battery depletion under diverse conditions. Compared to static baselines, it achieves 2.4× to 7.7× faster detection, maintains moderate energy consumption, and avoids system failure due to battery depletion across 125 deployment scenarios.

## 1. Introduction

Wildfires continue to cause severe damage to ecosystems, infrastructure, and human life worldwide. Although advances in satellite imaging, multi-sensor remote sensing, drone surveillance, and ground-based monitoring have improved fire tracking, early detection remains a persistent challenge, especially in remote or infrastructure-limited regions [1,2,3]. For example, the 2025 wildfire season in California and other global wildfires further underscored these limitations, with numerous fires intensifying uncontrollably and resulting in substantial environmental and economic losses [4,5].

Existing wildfire detection systems based on distributed sensors often rely on fixed sensing schedules, centralized cloud processing, or manually configured thresholds [6,7,8]. These designs are energy-intensive, prone to communication delays, and unsuitable for long-term deployment in resource-constrained environments. Furthermore, many approaches assume idealized conditions, overlooking the variability in weather conditions, solar energy availability, battery levels, and wireless connectivity-factors that critically impact real-world performance [6,9]. To address these limitations, wildfire detection systems must operate autonomously over extended periods, intelligently adapting their sensing and communication behaviors in response to dynamic environmental and energy conditions. Crucially, they must *balance early fire detection with energy conservation to remain operational without ongoing maintenance*.

While recent advances in sensing and machine learning have improved the accuracy of wildfire detection algorithms, deploying these systems in remote, real-world environments poses significant challenges. Remote sensors must operate autonomously for years in harsh environments without requiring maintenance, powered by solar energy and with limited battery capacity. Energy sustainability is difficult to guarantee due to substantial seasonal and daily variations in sunlight, energy leakage during idle periods, and unpredictable fire risks. Communication present is another bottleneck, as multi-hop wireless transmission consumes significant energy, and false alarms from smoke detection models can lead to unnecessary transmissions, thereby depleting the battery. In contrast, conservative smoke detection models may miss early smoke plumes, especially when weather conditions decrease visibility, increasing the risk of missing fires and delayed response. These competing requirements highlight the need for detection systems that are accurate, energy-aware, resilient to environmental conditions, and capable of autonomous long-term operation.

**Figure 1:**
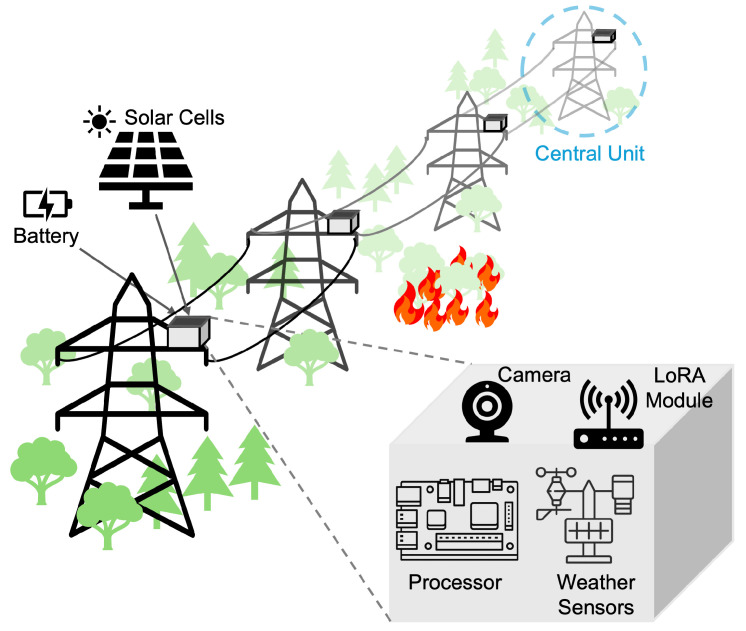
Overview of the EcoWild hardware platform. Each sensor node integrates a processor, weather sensors, a camera, and a LoRa module, powered by solar energy for long-term autonomous deployment.

This paper presents EcoWild, a cyber-physical system (CPS) designed for dynamic, energy-aware wildfire monitoring. We focus primarily on wildfires near high-voltage power transmission lines for two reasons. Wildfires near the power grid disrupt electricity transmission, causing significant disruptions [10]. Second, the power grid itself can trigger wildfires when the transmission lines come close to trees [11]. Therefore, the sensor suites considered in this work are placed in power towers, as demonstrated in Figure 1.

EcoWild integrates weather (temperature, humidity, anemometer) sensors, an RGB camera to detect smoke, an NVIDIA Jetson Orin Nano (NVIDIA Corporation, Santa Clara, CA, USA) [12] for real-time inference and control, and a LoRa communication module. It runs on a solar-powered embedded platform deployed on power towers in remote locations. A rechargeable lithium-ion battery is used for energy storage. The EcoWild uses a decision tree (DT) for fire risk estimation, lightweight on-device smoke detection (SD) models, and a reinforcement learning (RL) policy to guide adaptive sensing and communication. The vegetation, trees, and their density and types are also important factors [2,3]. Since these factors do not change dynamically, unlike the weather conditions, they are not used as runtime inputs. Instead, their impact is captured by the machine learning models trained for a given target location.

We employ two EcoWild sensor suite types: regular sensor suites and a gateway suite. In addition to detecting smoke and transmitting their alerts and images, the regular sensor suites also forward the alerts and images from their neighbors toward the gateway EcoWild. The gateway EcoWild has a cellular network with long-range uplink capability [13,14], so they can send their own data or the data forwarded by their neighbours directly to a control center.

The proposed EcoWild wildfire detection pipeline starts with sampling the weather sensors with a dynamically controlled sampling period. Instead of taking a picture and analyzing it in every interval, these inputs are first used to assess the risk using a decision tree (DT). This DT is trained on historical weather [15] and wildfire data [16] to accurately flag wildfire risk and save energy when the risk is low. If the DT indicates a high fire risk, EcoWild invokes smoke detection. Otherwise, it conserves energy by jumping to the fourth step to determine the next sampling period. If smoke or glow is detected, the potential wildfire is reported along with the image, weather data, and location to a decision-making center via a wireless sensor network. Since this step is energy-intensive, it is invoked only under high-risk conditions. Finally, a fixed sampling interval would waste energy if it is shorter than needed (during low-risk intervals) or increase detection time (during high-risk intervals). Therefore, our novel RL policy sets the next sampling period using recent sensor readings and outputs from the DT and smoke detection models to co-optimize the smoke detection time and battery energy level. This key contribution enables EcoWild to outperform approaches using a fixed sampling period.

To accurately account for energy, we incorporate solar energy harvesting profiles modeled with PVlib library [17] and detailed battery dynamics, including leakage and standby drain. Per-component energy costs for sensing, image capture, machine learning (ML) inference, RL decision-making, and LoRa communication are modeled based on empirical measurements and datasheet specifications. The system is trained and evaluated using wildfire imagery and synchronized weather logs from over 125 locations [16]. Historical weather logs, including temperature, humidity, and wind speed, are obtained from the Open-Meteo API [15] and aligned with ignition-labeled fire events to simulate realistic operating conditions. Our evaluations demonstrate that EcoWild consistently avoids battery depletion under field-representative deployment constraints. The following practical benefits, empirically validated across 125 real-world scenarios, highlight EcoWild ’s robustness, modularity, and energy efficiency:

- **Modular and Explainable Framework**: EcoWild is structured as a flexible pipeline where each component—DT-based risk estimation, smoke detection, and RL for the adaptive sampling—can be enabled or disabled independently. It supports any ML model that runs efficiently on edge devices, enabling customizable trade-offs between accuracy, energy, and responsiveness.
- **Dynamic and Adaptive Sensing**: The RL policy adjusts sampling periods in real time based on fire risk, battery level, and solar input, balancing responsiveness and energy conservation without requiring manual tuning.
- **Fully Embedded, Energy-Aware Operation**: All sensing, inference, and decision-making occur locally on solar-powered embedded devices, supporting long-term autonomy in remote, infrastructure-limited environments.
- **Robustness Across Deployment Scenarios**: EcoWild maintains reliable performance across seasonal and geographic variations under diverse communication conditions, including multi-node relaying and gateway-adjacent load.
- **Quantitative Advantages**: Compared to static policies, EcoWild achieves 2.4–7.7× faster wildfire detection with moderate energy consumption and no battery depletion.

The rest of the paper is organized as follows. Section 2 discusses related work. Section 3 details the system architecture, followed by the RL formulation in Section 4. Section 5.1 describes the dataset and simulation setup, and Section 5 presents our experimental evaluation. Finally, Section 6 summarizes our findings and outlines directions for future work.

## 2. Related Work

Wildfire detection research spans a range of application domains, including satellite imaging, UAV surveillance, and ground-based sensing. Recent advances incorporate embedded machine learning and reinforcement learning for adaptive sensing and control. While each approach contributes important capabilities, few systems address the whole challenge of long-term, autonomous wildfire detection in energy-constrained, remote environments.

Satellite-based detection provides broad-area coverage and has been widely used to detect fire via thermal anomalies or smoke plumes [9,18]. Recent remote sensing studies have advanced our understanding of wildfire dynamics by leveraging multi-source satellite imagery and spatiotemporal correlations. For instance, Tian et al. [2] use satellite-derived differenced normalized burn ratio (dNBR) and vegetation indices to analyze fire spread and post-burn vegetation recovery. Similarly, Dadkhah et al. [3] examine the long-term wildfire–climate interactions using burned area, land use/surface temperature, and normalized difference vegetation index (NDVI) products of moderate resolution imaging spectroradiometer (MODIS) as well as the Climate Hazards Group Infrared Precipitation with Stations (CHIRPS) datasets. While these advances provide valuable insights into wildfire–climate interactions and vegetation recovery, they remain constrained by coarse resolution, limited revisit frequency, and dependence on cloud-free imagery, making them less suitable for continuous, fine-grained monitoring near power infrastructure—the focus of our CPS-based approach.

UAV-based detection offers greater flexibility and spatial precision, enabling high-resolution imaging of wildfire-affected regions [19]. Nevertheless, UAVs face significant limitations, such as short flight durations, the need for frequent recharging, and operator supervision—making them unsuitable for continuous, unattended monitoring in large-scale deployments.

Ground-based detection has also been explored for early fire detection using temperature, humidity, or smoke sensors [8,20]. These systems typically use wireless sensor networks to offer real-time monitoring and even forecast fire danger levels through in-network processing. However, they generally lack wide-area coverage and may require integration with visual systems for comprehensive situational awareness. To complement these limitations, vision-based and ML-driven methods have gained traction with the advent of machine learning. For example, SmokeyNet [16] uses high-resolution imagery and deep CNNs for smoke classification. However, such models are typically deployed in cloud environments and require substantial computational resources. Xyloni [21] proposes a low-power accelerator for on-device inference using Shallow CaffeNet, but it lacks dynamic sensing control and may sacrifice accuracy for efficiency. One such visual approach is proposed by Ding et al. [9], which uses deep learning on image data from ground cameras to detect wildfires in remote forests. While this method enables visual confirmation and long-range communication via LoRa, it still assumes stable power availability, making it less suitable for long-term autonomous deployments.

Reinforcement Learning has increasingly been explored in energy-efficient embedded systems to optimize wildfire sensing and coordination behavior. Tuncel et al. [22] have proposed an RL-based cyber-physical system that dynamically adjusts sensor sampling intervals to extend operational lifetime in wildfire monitoring scenarios. While effective in energy management, this system lacks vision-based inference tailored to wildfire detection and relies on simulated rather than real-world weather data. RL has also been used in wildfire-adjacent domains for planning and coordination. ForestProtector [23] and Julian & Kochenderfer [24] apply RL to optimize UAV trajectories and sensor placements, but these systems assume idealized energy availability and lack runtime adaptability—making them unsuitable for continuous, embedded deployments. More recent frameworks, such as PyroTrack [25] and Diaz et al.’s Twin Delayed Deep Deterministic Policy Gradient (TD3)-based UAV swarm coordination [26], incorporate battery constraints and communication costs into multi-agent RL control. However, these approaches are designed for mobile agents, not static, solar-powered sensor nodes. They also lack the fine-grained environmental adaptability and embedded vision integration required for sustainable wildfire monitoring in real-world, resource-constrained settings.

A key limitation of existing work is the lack of integration across sensing, inference, and energy management, which undermines adaptability and long-term sustainability in real-world deployments. Our prior work [27] proposed a static optimization framework for wildfire detection under energy constraints. However, it employs a fixed sampling period policy (used as a baseline for comparison in this work). Moreover, it does not account for dynamic weather and battery conditions and relies solely on statistical data without dynamic simulation. In contrast, the proposed EcoWild framework integrates decision tree-based fire risk estimation, lightweight smoke detection, and reinforcement learning for dynamic sensing control. Unlike earlier methods that treat detection, control, or energy modeling in isolation, EcoWild jointly optimizes all components while modeling solar harvesting, battery dynamics, and multi-node communication—enabling sustainable operation in dynamic, real-world conditions.

## 3. EcoWild Framework

**Figure 2:**
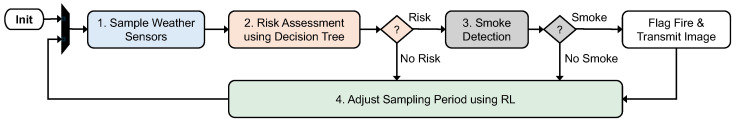
The proposed EcoWild framework starts by sampling temperature, humidity, and anemometer sensors. A decision tree assesses wildfire risk using these inputs. If necessary, the system captures and analyzes a new image using a smoke detection model. If smoke is detected, a potential fire is reported along with the image, weather data, and location. The RL policy monitors all operations and adjusts the sampling period to co-optimize wildfire detection time and battery energy usage.

EcoWild operates through a multi-stage, closed-loop perception-action cycle, which organizes the proposed wildfire detection pipeline into five coordinated and explainable modules, as illustrated in Figure 2. This section describes the details of this pipeline.

### 3.1. Sample Weather Sensors

At the beginning of each sampling period, EcoWild activates the temperature, humidity, and anemometer sensors. The RL agent determines the sampling period in the previous sensing interval based on the current battery level, solar input, and recent system history, after which the system samples the weather sensors once the interval elapses. These sensor readings feed in to the DT classifier for risk assessment.

### 3.2. Risk Assessment Using a Decision Tree

We train a lightweight DT model using historical weather data [15] and wildfire data [16]. This DT assesses the wildfire risk at runtime using the current sensor inputs. The DT captures the well-established correlation between high temperatures, low humidity, and the risk of wildfire, achieving a true positive rate of 96%. This high sensitivity ensures that potential fire conditions are rarely missed. The probability of failing to detect a fire across two consecutive intervals is only 0.0016. To achieve this level of sensitivity, the DT has a 34% false positive rate, which is acceptable at this stage because subsequent image-based smoke detection modules validate the assessed risk before transmitting the wildfire alerts and images. If the DT indicates a high risk of wildfire, EcoWild activates the smoke detection module; otherwise, the system conserves energy by skipping it and determines the next sampling interval. In addition to its effectiveness, the DT is modular and computationally lightweight, enabling periodic retraining to account for seasonal or regional variations in weather patterns, thereby supporting long-term and localized wildfire risk estimation.

### 3.3. Smoke Detection

When the DT model assesses a high-risk wildfire condition, EcoWild activates the camera to capture an RGB image of the surrounding environment. The image is processed locally on the edge device using a lightweight smoke detection model designed to handle both daytime smoke and nighttime glow.

Specifically, EcoWild employs an ensemble of ResNet34 [28] and YOLOv8 [29] models, combined under an OR-based decision rule, so that a frame is classified as smoke-positive if either model detects smoke. This ensemble approach improves robustness while maintaining efficient inference on resource-constrained hardware. We apply both whole-frame and tiled image analysis to enhance detection accuracy and spatial coverage further. In the whole-frame method, the system evaluates the image directly. In contrast, in the tiled method, the image is divided into overlapping 640 × 640 pixel tiles with a 10% margin, allowing the system to detect small or amorphous smoke regions that might be missed in the full-frame view. If any tile is classified as smoke-positive, the entire image is flagged as having fire. Although this work focuses on the ResNet34–YOLOv8 ensemble, EcoWild is designed to remain modular, supporting alternative smoke or fire detection models optimized for embedded platforms such as the Jetson Orin Nano [12].

### 3.4. Communication Decision

EcoWild operates independently and triggers wireless LoRa communication only when it detects smoke. If no smoke is detected, the sensor suites conserve energy by entering low-power sleep mode until the next sensing cycle, determined by the RL policy described in Section 4. When smoke or glow is detected, EcoWild reports the potential wildfire—together with the captured image, relevant weather data, and the sensor node’s location—to a decision-making center via the wireless sensor network.

   We employ two EcoWild sensor suite types:

- **Regular sensor suite:** These EcoWild sample the weather sensors and operate as discussed so far. In addition to transmitting their own wildfire alerts and images, they also forward the alerts and images they receive from their neighbors toward the gateway suites.
- **Gateway sensor suite:** In addition to all hardware in the regular suites, the gateway EcoWild has a cellular network with long-range uplink capability [13,14]. In this way, they can send their own data or the data forwarded by their neighbors directly to a control center. They are placed intermittently (e.g., every $N_{S}$ towers) since they require extra communication hardware and experience the highest communication burden.

The simple line topology along the power towers simplifies the routing. Since each sensor suite is at most $\left\lceil N_{S}/2\right\rceil$ hops away ($N_{S}=5$ in this work), each regular suite forwards the data to its immediate neighbor toward the closest gateway sensor suite to minimize the communication energy cost.

## 4. Energy-Aware Sensing Scheduling with RL

Frequent sensing enables timely wildfire detection but consumes more energy and shortens the battery lifetime. Static schedules fail to adapt to changing environmental risk or battery conditions, as demonstrated in Section 5. To overcome this, EcoWild formulates sensor scheduling as a reinforcement learning problem, where an agent learns to dynamically select sensing intervals that balance energy sustainability with detection responsiveness in solar-powered embedded deployments. This key contribution enables EcoWild to outperform approaches using a fixed sampling period. It decreases the sampling period to assess wildfire risk more frequently when risk is high. In contrast, it increases the sampling period to let the system sleep longer and save energy, depending on the wildfire risk and battery level.

### 4.1. Overview of the Proposed RL Technique

Reinforcement learning enables an agent to learn to maximize cumulative rewards by interacting with the environment. It is typically modeled as a Markov Decision Process (MDP), defined by the tuple M=(S,A,P,R,γ). Here, S denotes the state space of the environment, and A is the action space available to the agent. *P* is the transition probability function, describing how the environment evolves in response to the agent’s actions. *R* is the reward function, and γ∈[0,1] is a discount factor that exponentially reduces the importance of future rewards. At time step *t*, the RL agent observes the state $s_{t}\in S$ that reveals current weather information, energy status, and past decisions. It then takes the decision $a_{t}∼\pi \left(\cdot |s_{t}\right)$ and receives reward $r(s_{t},a_{t})$. In our work, state transition is controlled by a high-fidelity wildfire simulator, described in Section 5.1. The interactions with the environment are then used to maximize the expected cumulative reward:

$$J\left(\pi \right)=E_{\pi }\left[\sum_{t=0}^{\infty }\gamma^{t}r\left(s_{t},a_{t}\right)\right]$$

The following subsections define the agent’s state space, action space, reward formulation, learning algorithm, and deployment setup.

### 4.2. State and Action Spaces

The state space $S\subset R^{11}$ consists of 11-dimensional vectors that encode key information for the agent’s decision-making process. The elements in S can be divided into three categories. The first category captures environmental context, including weather sensor readings, date, and time. The second category includes data on energy harvesting and battery level. The third category represents the agent’s previous decisions and image classification outcomes, as summarized in Table 1. Together, these components provide the agent with rich information to evaluate the risk of wildfire and support intelligent trade-offs between early-fire detection and energy usage, ultimately enabling optimal control of the next sampling period.

The action of the agent controls the sampling period, which determines when the system takes and processes the next sample. The action space is one-dimensional and represented by the interval $\left[t_{\min},t_{\max}\right]$. For ease of simulation, the action $a_{t}\in A$ is discretized to the nearest integer. Although the framework supports a configurable range, this work restricts the interval to $t_{\min}=1$ min and $t_{\max}=30$ min.

### 4.3. Reward Function Design

The reward function represents the optimization goal of the reinforcement learning problem and has three components. First, it encourages early detection by assigning higher rewards for detecting fires earlier. Second, it imposes a large negative reward for depleting the battery. Both fire detection and energy depletion trigger termination of the episode, so the first two rewards are given at the end of each episode. While these end-of-episode rewards are theoretically sufficient to define the optimization objective, their sparsity makes it difficult for the agent to converge. To address this, a third, step-based reward is introduced to guide the agent in adjusting its sampling period based on the DT output. This step-based reward is intentionally kept small relative to the first two, as it does not directly serve the main optimization goal.

**End-of-Episode Reward:** At the end of each episode, a final reward $r_{\mathrm{end}}$ is calculated based on the system’s energy outcome:

**Case 1 (Battery is depleted):**

(1) $$\begin{array}{cc} r_{\mathrm{end}}=-\alpha_{B}\frac{1}{t_{\mathrm{deplete}}-t_{\mathrm{start}}}-R_{\min} & \end{array}$$

where $t_{\mathrm{deplete}}$ is the battery depletion time and $t_{\mathrm{start}}$ is the episode start time. This strongly penalizes unsustainable policies. The reward becomes increasingly negative as the depletion time $t_{\mathrm{deplete}}$ approaches the start of the episode, strongly discouraging early battery exhaustion.

**Case 2 (Battery is not depleted):** The agent accumulates a low-pass filtered reward over the episode: $r_{t}=\beta \cdot t_{\mathrm{lastSampling}}+\left(1-\beta \right)\cdot r_{t-1}$, and the final reward is:

(2) $$r_{\mathrm{end}}=-k\cdot r_{t}$$

where β and *k* are tunable parameters to encourage consistently lower sampling periods without battery exhaustion.

**Step-Based Reward:** After each sensing action, a small step reward $r_{\mathrm{step}}$ is assigned based on the last sampling period $t_{\mathrm{lastSampling}}$ and the estimated fire risk predicted by a DT:

(3) $$r_{\mathrm{step}}=t_{\mathrm{lastSampling}}\left(1-2\cdot \mathrm{DT}\right)$$

where DT∈{0,1} indicates the DT prediction of low or high wildfire risk. This reward structure encourages the agent to increase sensing frequency under high-risk conditions and conserve energy under low-risk conditions. When the risk is high (e.g., DT=1), the reward is negative and is maximized by decreasing the sampling period. Conversely, when DT=0, the agent increases the sampling period to maximize the reward. It is important to note that this reward is designed to facilitate stable convergence of the RL algorithm rather than serve as the primary optimization objective. To reflect this, the step-based reward is normalized by the total number of samples, ensuring its magnitude remains small relative to the terminal end-of-episode rewards.

**Energy-Aware Risk Adaptation:** High wildfire risk conditions often coincide with high-temperature periods, which also enable greater solar energy harvesting. EcoWild’s reward design explicitly incorporates this relationship: during high-risk intervals, when solar input is also likely high, the agent is rewarded for adopting shorter sensing intervals to enable faster detection. This encourages the agent to exploit favorable energy conditions when responsiveness is most critical, while conserving energy during low-risk periods.

### 4.4. Learning Strategy

The RL agent is trained using the Twin Delayed Deep Deterministic Policy Gradient (TD3) algorithm [30], which is well-suited for our problem due to its ability to handle continuous action spaces and its improved training stability. We implement TD3 using the Stable-Baselines3 library [31], a widely used framework that offers modular and reliable reinforcement learning algorithms built on top of PyTorch 2.3.0 and compatible with OpenAI Gym environments. TD3 addresses common issues such as overestimation bias and high variance through techniques including twin Q-networks, target policy smoothing, and delayed policy updates, making it a strong candidate for learning stable policies in our environment. The actor and critic networks are implemented using a multilayer perceptron (MLP) architecture provided by the MlpPolicy in Stable-Baselines3, which uses fully connected layers suitable for low-dimensional state spaces. Our key enhancements in this work include:

**Action Noise Scaling:** We apply linearly annealed Gaussian noise to the TD3 action outputs to encourage exploration during early training and later stabilize policy convergence. The action noise starts with a magnitude equivalent to 5 min and decreases linearly to 1 min by the midpoint of training. After this point, a constant 1-min noise is maintained for the remainder of the simulation to support stable fine-tuning. This annealing scheme ensures early-stage exploration while avoiding later-stage erratic behavior.

**Figure 3:**
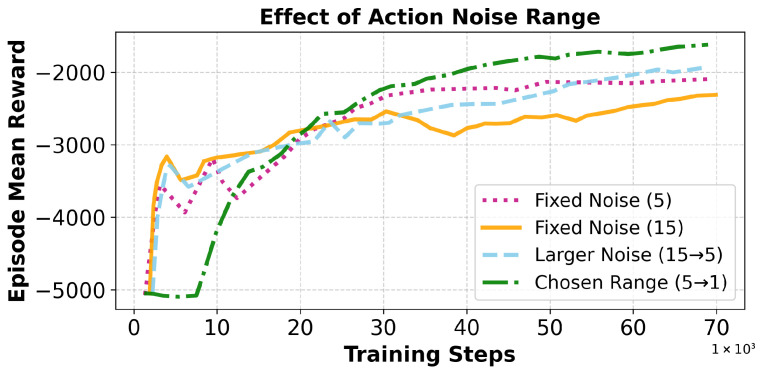
Ablation study of different action noise ranges. Fixed noise schedules at 5 and 15 min either show instability or plateau at lower rewards. A larger range (15 → 5 min) exhibits oscillations. The chosen range (5 → 1 min) achieves stable convergence and the best final performance.

To encourage sufficient exploration while ensuring noise does not corrupt effective actions, we set the action noise upper bound to 5 min. This limit is consistent with both our target wildfire detection window (≤5 min) and the default parameter for the TD3 algorithm [30]. The lower bound of 1 min reflects the fastest feasible sampling period in our hardware setup and matches the minimum of the action space. Annealing noise from 5 → 1 min, therefore, covers the entire practically useful exploration range while avoiding unstable jumps outside feasible intervals. To complement this rationale, we performed an ablation study (Figure 3) comparing four schedules: (i) Fixed noise (5 min), which converges but shows instability in early training; (ii) Fixed noise (15 min), which converges slowly and plateaus early at a lower reward; (iii) a larger range (15 → 5 min), which shows oscillations and unstable convergence; and (iv) our chosen range (5 → 1 min), which converges smoothly and achieves the best final performance. This evidence confirms that the 5 → 1 min annealing range achieves both stable training and strong final results, justifying our parameter selection.

**Reset Mechanism:** During training, we prevent the agent from converging to a local optima that consistently selects extreme sampling periods—either very long (e.g., close to 30 min) or very short (e.g., close to 1 min)—over multiple episodes. These behaviors are undesirable since long intervals delay fire detection, while excessively short intervals waste energy. We prevent this behavior using a policy reset mechanism. If the agent repeatedly selects extreme sampling periods for a fixed number of consecutive episodes, we reset the actor and critic network weights using Xavier initialization [32]. This promotes renewed exploration and helps the agent escape suboptimal policies. Importantly, only the policy networks are reset—optimizer state and replay buffer are preserved to retain previously gathered experience and avoid complete relearning.

Without the reset mechanism, we observed that approximately one out of every five seeds (about 20%) collapsed into extreme behavior, where the policy selected either very high or very low sampling intervals for the entire run and never recovered. Importantly, when this collapse occurred, the chosen sampling intervals showed no meaningful correlation with the system state (e.g., temperature, battery level, HDWI score). In other words, the policy acted inconsistently with the input features and produced nonsensical decisions. Through empirical analysis, we also found that if such extreme behavior continued for four or more consecutive episodes, the policy never returned to normal operation. This observation motivated our reset rule: the policy is reinitialized if collapse-like behavior persists for four back-to-back episodes. With this mechanism, none of the seeds exhibited collapse across all runs. This quantitative evidence (about 20% collapse rate without reset, none with reset) supports the choice of the four-episode threshold. It also demonstrates that the reset function effectively prevents the policy from stacking in local optima.

**Training and Deployment:** We train the TD3 agent offline using historical weather data from Open-Meteo [15] and solar irradiance traces generated by PVlib [17]. Each episode simulates a real sensor’s behavior, including minute-level energy harvesting, sensing, communication, and battery leakage. The agent interacts with this environment by choosing sampling periods and observing their effects on fire detection timing and energy sustainability. Once training converges, the final TD3 policy is exported and deployed onto embedded hardware in inference-only mode. The deployed policy maps real-time sensor inputs to sampling decisions without online updates, ensuring low computational overhead and reliable behavior under constrained energy budgets.

## 5. Experimental Results

### 5.1. Experimental Setup

EcoWild is designed for autonomous sensing, inference, and wireless communication using the following components:

- Weather sensors, an SHT10 temperature and humidity sensor [33], a Davis DS6410 anemometer [34], for monitoring atmospheric conditions relevant to fire risk.
- A Sony IMX219 8-megapixel RGB camera [35] to take environmental images for daytime smoke detection and nighttime fire or glow detection.
- A NVIDIA Jetson Orin Nano [12] embedded device for real-time, on-device inference and adaptive decision-making using reinforcement learning and risk estimation.
- A LoRa radio module [36] for long-range, low-power wireless communication.
- A solar panel [37] and rechargeable battery for continuous energy harvesting and storage. The panel consists of 24 cells and has specifications of 6 V and 2.38 W at the maximum power point, with 21.5% efficiency. We target long-term, maintenance-free operation by dynamically adapting sensing and communication schedules based on real-time battery levels, sunlight availability, and fire risk—ensuring sustainable energy use without requiring manual recharging or battery replacement [38,39,40,41].

**Dataset and Offline Logs:** To enable realistic, repeatable, and data-driven evaluation, EcoWild leverages datasets constructed from real-world environmental, operational, and wildfire sources with the following modalities:

- *Weather and Environmental Logs:* Historical temperature, humidity, and wind speed data are collected for each camera location using the Open-Meteo archive API [15]. Weather data goes back up to one year prior to image collection and fire start time in one-minute granularity.
- *Smoke Image and Fire Event Labels:* Smoke ignition events are sourced from the public FigLib wildfire dataset [16], which provides time-sequenced images from multiple camera locations. Each location contains 81 images captured at 1-min intervals: 40 images with no smoke, followed by one ignition event, and 40 post-ignition images containing smoke. The dataset is partitioned into 70% training, 15% validation, and 15% testing splits, following the standard configuration used in prior work [27]. Ground-truth fire labels are aligned to the ignition frame for each location to support supervised RL training.
- *Solar Energy Data:* Solar panel energy harvesting is simulated at each location using the PVlib library [17] and a single-diode photovoltaic model calibrated to a UV-resistant 6 V, 2.38 W panel [37]. Hourly solar irradiance profiles are generated based on the GPS coordinates of the camera sites provided in the FigLib dataset [16], then interpolated to 1-min granularity. The panel azimuth was set to 180° and the tilt angle to the site latitude (34°) to maximize the annual average energy yield. The solar model incorporates temperature effects, soiling losses, and wiring inefficiencies to reflect realistic panel behavior.

Each log entry in the dataset consists of a timestamped environmental state vector (weather features, solar energy input, battery status) and the corresponding wildfire label. These logs provide the RL agent with minute-by-minute environmental variability grounded in real-world geographic and temporal conditions.

**Power/Energy Models:** The simulation environment models the full energy pipeline for sensing, processing (including decision tree evaluation and smoke detection inference), and communication, as follows:

- *Active Energy Consumption:* We account for the active energy cost of each operation, including weather sensing, image capture, decision-making, SD inference, RL inference, and LoRa-based communication. These values are derived from empirical measurements on embedded hardware platforms, as detailed in our prior work [27]. Component-specific characterization includes the SHT10 temperature and humidity sensor, for which active power consumption is obtained from the manufacturer’s datasheet [33]; the DS6410 anemometer, a passive sensor whose energy usage depends on microcontroller pulse processing, following the method described in [42]; and the LoRa transceiver (STM Nucleo-WL55JC2), where transmission energy was measured and standby draw is based on datasheet specifications [36]. The camera’s active energy was empirically measured, while its standby power is derived from literature [43].
- *Standby and Leakage Losses:* Standby energy drain from all hardware components, along with battery self-discharge, is incorporated into the simulation’s energy model. Standby values for the SHT10 temperature-humidity sensor and LoRa transceiver are taken from respective datasheets [33,36], while the camera’s standby consumption is obtained from prior literature [43]. Battery leakage is modeled using conservative estimates from published work, assuming a low self-discharge rate below 5% per month [44].
- *Deployment-Aware Energy Reserve and Losses:* To reflect real-world deployment constraints, we provision each sensor suite with a 7-day battery energy reserve, ensuring uninterrupted operation during extended periods of low solar irradiance (e.g., overcast days or shaded locations). Additionally, we model realistic solar harvesting losses due to environmental factors such as dirt accumulation, panel tilt, and shading. In our simulations, we assume a 50% harvesting loss for edge sensor suites (typically at the network perimeter with limited solar exposure), and a 30% loss for relay and gateway-adjacent suites. These deployment-aware assumptions ensure that EcoWild remains robust under practical conditions where harvested solar energy may be significantly reduced.

This structure allows the RL agent to interact with a realistic, temporally-aligned simulation environment, where energy constraints, environmental variability, and fire event timing are grounded in real-world conditions.

**Smoke Detection Models:** We utilize smoke detection models using ResNet34 [28] and YOLOv8 [29] networks trained on the FIgLib dataset [16]. We evaluated their true positive rate (TPR), false negative rate (FNR), false positive rate (FPR), and true negative rate (TNR), exhaustively in our prior work [27]. The TPR reflects the system’s ability to correctly detect actual fire events, while the FNR captures the frequency of missed fires. FPR quantifies unnecessary fire alerts, which result in energy waste, and TNR measures how reliably the system identifies non-fire scenarios. Our framework emphasizes minimizing FNR to ensure fires are not missed, reducing FPR to conserve energy, and maintaining high TPR and TNR for consistent, dependable operation in energy-constrained environments. We utilize two variants of the smoke detection model to study the trade-off between detection speed and energy consumption, listed in Table 2. The aggressive performance model prioritizes fast detection at the expense of more false alarms and energy use. In contrast, the conservative (low energy) model reduces communication overhead by being selective in its predictions. We emphasize that even the conservative model guarantees eventual fire detection, since the probability of missing a fire after *n* time steps is $1-{\left(1-TPR\right)}^{n}$ [27].

### 5.2. Static Baseline Algorithms Used for Quantitative Comparisons

We compare the proposed EcoWild framework against several static algorithms, which are used with a fixed sampling period, to evaluate the effectiveness of the proposed algorithm with a dynamic sampling period determined by RL. The static algorithms used as baselines are constructed by selectively enabling or disabling key system modules: DT-based risk estimation, smoke detection, and reinforcement learning. This modular design enables us to isolate the contribution of each component and better understand its individual and combined impact.

- **Fixed baseline** captures weather data and images at every fixed interval and transmits them without any local filtering, decision-making, or smoke detection.
- **DT-only algorithm** uses the same DT used in EcoWild to evaluate wildfire risk from weather data. An image is captured and transmitted only when the estimated risk is high *without running the smoke detection algorithm*.
- **SD-time algorithm** takes an image at each interval (*without a DT*) and performs smoke detection using the aggressive performance SD model (see Table 2). This smoke detection-based filtering prioritizes fast detection but leads to increased communication and energy consumption.
- **SD-energy algorithm** is the same as the SD-time algorithm (i.e., takes and processes images at every interval), but it uses the conservative (low energy) SD model. It minimizes the communication and energy use, at the potential cost of delayed detection.
- **DT-SD-time algorithm** combines the DT-based wildfire risk estimation and aggressive ML-based smoke detection (see Table 2). The DT filters out low-risk intervals, and the SD model further refines image transmission decisions by prioritizing fast detection under high-risk conditions.
- **DT-SD-energy algorithm** performs like the DT-SD-time algorithm, but it uses the conservative (low energy) SD model (see Table 2). This configuration minimizes communication and energy usage while still detecting probable fire events.

Table 3 summarizes the configuration of each baseline algorithm and highlights how EcoWild uniquely integrates all key components—fixed sensing, decision tree risk estimation, smoke detection, and reinforcement learning.

To evaluate EcoWild’s generalization across diverse deployment scenarios, we simulate wildfire detection at 125 sensor suite locations throughout California [16]. We focus on California because it provides the only large, publicly available wildfire smoke image dataset needed to train and evaluate our ML-based smoke detection models. Comparable labeled datasets are unavailable in other climate zones, making direct evaluation infeasible. To address generalizability beyond the Mediterranean climate of California, we incorporate deployment-aware assumptions: specifically, we apply 30–50% harvesting losses to emulate energy scarcity and enforce a 7-day battery reserve to reflect prolonged low-sunlight conditions. These stress-test conditions mimic the challenges that would arise in boreal, tropical, or arid climates without requiring new image datasets. The results show that EcoWild maintains sustainable operation and low-latency fire detection even under such harsher conditions. Each location participates in a multi-node communication structure, where intermediate suites may relay messages before reaching a gateway, as detailed in Section 3.4. We assess EcoWild’s performance along multiple dimensions, including adaptability, energy efficiency, and detection responsiveness, and compare it against a suite of fixed-interval baseline policies.

### 5.3. Balancing Responsiveness vs. Sustainability

To balance detection responsiveness with energy sustainability, EcoWild employs a reward function that integrates per-step and end-of-episode feedback. The full reward formulation is detailed in Section 4.3, where Equation (3) defines the step-based reward based on estimated wildfire risk, and Equations (1) and (2) define the terminal rewards for battery depletion and safe operation, respectively. Tunable parameters weight these components to balance early fire detection with long-term energy preservation.

We performed a hyperparameter sweep on β∈{0.1,0.2,…,1.0} to explore different trade-offs between energy and responsiveness. Based on empirical performance across locations, we selected the values listed in Table 4 to ensure reliable fire detection without depleting battery reserves prematurely. These values were selected to ensure that EcoWild maintains high average battery levels while keeping detection time under 5 min in most conditions. While the agent can adapt to different risk and solar scenarios, tuning these reward weights was essential for robust generalization across locations. Specifically, $\alpha_{B}$ =525,600 appears in the battery depletion penalty (Equation (1)) and reflects the total number of minutes a year, ensuring that early depletion is heavily penalized. $R_{\min}=5000$ is also used in Equation (1) to impose a fixed penalty for unsustainable behavior. We chose β=0.9 (used in Equation (2)) to control the smoothing of the sampling interval penalty, balancing responsiveness and energy preservation. Finally, k=100 scales this smoothed penalty in the same equation, increasing its impact relative to step-based rewards and helping the agent avoid inefficient sampling behavior.

### 5.4. Risk-Aware Sampling Behavior

**Figure 4:**
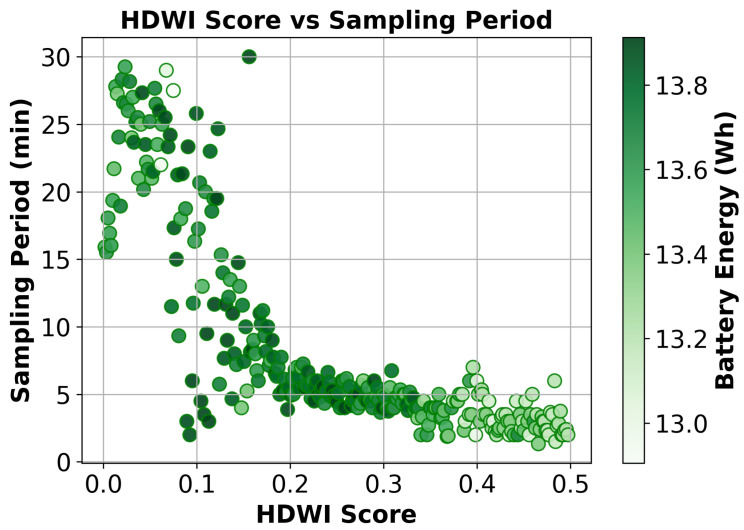
Correlation between HDWI [45] risk score and the sampling period. Under high risk (high HDWI), EcoWild reduces the sampling period for faster detection. Point color represents battery energy at the time of sampling.

We validate that EcoWild learns meaningful control policies by analyzing how sensing decisions correlate with estimated fire risk. Figure 4 shows the sampling period as a function of the Hot-Dry-Windy Index (HDWI) [45], a widely used fire risk metric derived from temperature, humidity, and wind speed. Each point represents a sensing decision, with color indicating the battery energy (in Wh) at that moment.

EcoWild exhibits a clear inverse correlation between HDWI [45] and sampling period. Figure 4 shows that low HDWI scores (low fire risk) correspond to larger sampling periods (less frequency sensing). As expected, the sampling period decreases to sense more frequently as the HDWI score (i.e., the wildfire risk) increases. The Pearson correlation coefficient is found as −0.79, confirming the negative correlation. The agent selects short sampling periods (1–5 min) under high-risk conditions (HDWI >0.25), to enable faster detection. In contrast, under low-risk conditions (HDWI <0.2), which account for approximately 55% of the data, the agent conserves energy by sampling less frequently (15–30 min). This behavior is particularly beneficial during cold or humid seasons, where fire likelihood is low, and battery preservation becomes critical. Battery levels remain stable and consistently over 13 Wh during aggressive sampling, indicating that EcoWild balances responsiveness with long-term sustainability through risk-aware adaptation.

### 5.5. Multi-Node Evaluation and Sustainability Analysis

**Figure 5:**
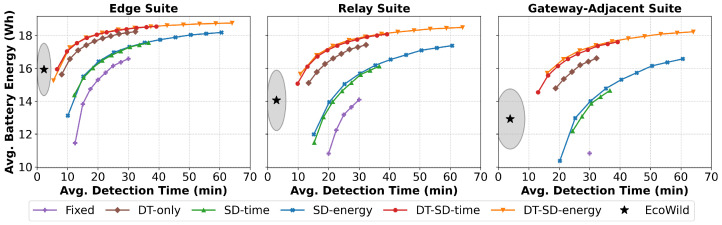
Average detection time vs. battery energy across a sensor suite furthest from the gateway (edge), another one midway to the gateway (relay), and a gateway-adjacent sensor suite. The communication energy burden increases since more images and data are forwarded by sensor suites closer to the gateway suites. **EcoWild (black star)** achieves fast detection and sustainable energy usage across all configurations, outperforming all fixed baselines.

This section evaluates EcoWild against a suite of fixed-interval baselines introduced in Section 5.2, under three representative communication configurations defined in Section 3.4. The first sensor suite (edge) is the furthest away from a gateway, so it does not need to forward data from other sensor suites. The second (relay) and third (gateway-adjacent) sensor suites forward more images and data besides their own data, increasing their communication energy burden. These configurations reflect varying forwarding responsibilities across sensor suites and capture the increased communication energy based on node placement and network topology. Key Observations from Figure 5 are as follows:

- Superior detection time in all scenarios: EcoWild (black star) consistently outperforms the best-performing configuration of each baseline, achieving 2.4–7.7× faster detection while maintaining moderate energy use.
- Pareto Frontier Breaker: Baseline policies provide a visible Pareto trade-off between detection time and energy. EcoWild lies outside this frontier in all three settings, demonstrating its ability to achieve both goals simultaneously.
- Widening Advantage in High-Cost Settings: As additional communication energy burden increases, the performance gap between EcoWild and the baselines becomes more pronounced, especially for sensor suites close to the gateway that need to forward more messages from their neighbors.
- Sustained battery energy: EcoWild (black star) never depletes the battery energy in any of the considered scenarios in 125 locations. It maintains an average battery energy of 6 Wh (edge), 14 Wh (relay), and 13 Wh (gateway-adjacent), never dropping below 11 Wh in any scenario.

**Figure 6:**
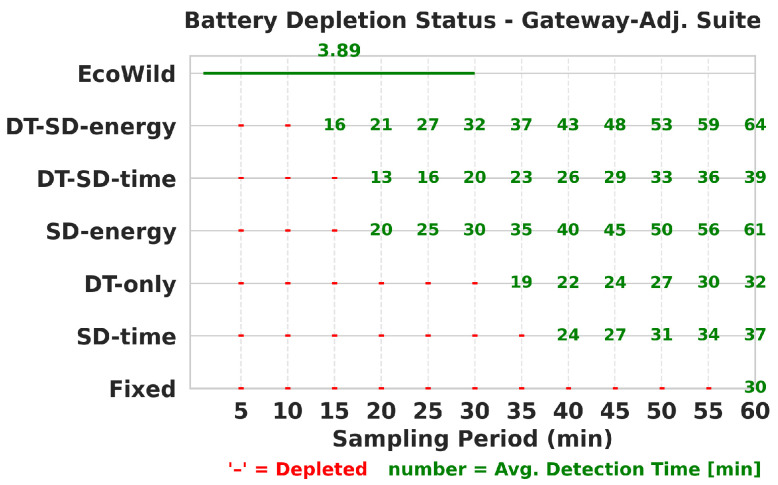
Battery depletion under gateway-adjacent configuration. Red dashes indicate energy depletion; green numbers indicate average detection time. EcoWild avoids depletion and achieves 3.89-min detection time.

We further analyze battery depletion behavior to validate these trade-offs under the most demanding conditions. Figure 6 shows that fixed baselines deplete rapidly under aggressive sampling periods. In contrast, EcoWild maintains sustainable operation across all test seeds—even in more demanding conditions—while achieving fast average detection (under 5 min). This highlights EcoWild’s real-world deployability in energy-constrained multi-node settings.

### 5.6. Per-Location Comparison: Generalizability

**Figure 7:**
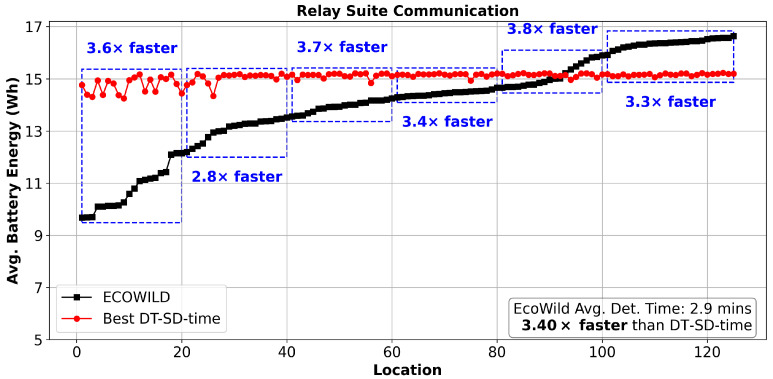
Per-location comparison of average battery energy under realistic multi-node communication. While EcoWild (black) consistently outperforms the best baseline (DT-SD-time, red) in detection time across all 125 deployments, its battery energy is slightly lower in some locations—yet remains above critical thresholds, demonstrating sustainable operation.

This section analyzes the performance of EcoWild at each of the 125 deployment locations in more detail. Figure 7 compares EcoWild (black) against the best-performing fixed baseline (DT-SD-time, red) in terms of average battery energy across locations. EcoWild achieves an average detection time of just 2.9 min—3.40× faster than the DT-SD-time baseline—while consistently maintaining battery levels well above critical thresholds across all deployment locations. The energy differences are especially notable in high-load relay or gateway-adjacent suites, where fixed baselines consume more energy due to continuous message forwarding. At the same time, EcoWild sustains comparable or moderately lower battery levels with significantly faster detection. Overall, the observed performance spread across locations highlights EcoWild ’s ability to adapt dynamically to diverse environmental conditions and communication roles, in contrast to the rigid, one-size-fits-all behavior of fixed policies. These results confirm that EcoWild generalizes well across heterogeneous deployments without requiring manual configuration or location-specific tuning.

### 5.7. Limitations and Future Work

EcoWild outperforms static baselines by up to 7.7× in detection latency while maintaining sustainable energy usage. It detects fires by activating weather and camera sensors at a dynamically adjusted rate and detecting smoke in images. These results confirm that dynamic policies outperform fixed-rate sampling in both responsiveness and longevity. EcoWild builds on this foundation by explicitly modeling solar harvesting, battery leakage, and communication costs, extending existing frameworks to operate under more realistic and variable energy conditions.

Despite its promising results, EcoWild has several limitations. First, our evaluations focus on 125 locations in California due to the availability of the FigLib dataset, which limits geographic generalizability. Similarly, we analyze three representative communication configurations across a network of 125 sensor suites. EcoWild can detect fires in large, forested areas if the sensor suites on power towers view the smoke. However, system-wide evaluation of the collective behavior of all sensors that cover a complete power grid remains to be validated. Third, while our simulation models incorporate solar, battery, and environmental dynamics, they abstract away interactions between nearby nodes, such as contention, interference, or coordinated detection opportunities. Uncertainties in our data and evaluations stem primarily from (i) the variability and resolution of real-world solar and weather data, (ii) assumptions made in energy modeling (e.g., fixed energy leakage rates, linear discharge profiles), and (iii) simplifications in vision-based smoke detection (e.g., thresholding parameters). While these are reasonable for simulation-scale evaluation, they must be revisited during field deployments.

To strengthen EcoWild’s robustness and generalizability, future work should extend EcoWild to deployments in other geographic regions and climate zones, once labeled wildfire imagery from those environments becomes available. The evaluation should also integrate vegetation and fuel type data for higher accuracy. We also plan to extend EcoWild to support fully distributed multi-hop coordination, enabling collaborative decision-making across sensor nodes. This includes cross-node energy balancing, congestion-aware communication, multi-sensor fusion, and exploration of shared battery state to inform forwarding strategies. Finally, pilot-scale dense field deployments are needed to further to assess EcoWild’s performance in the real world.

## 6. Conclusions

This paper presented EcoWild, a reinforcement learning-based cyber-physical system for energy-aware wildfire detection in remote environments. EcoWild integrates decision tree-based fire risk estimation, lightweight smoke detection models, and adaptive sensing policies trained via reinforcement learning. The system is deployed on solar-powered embedded hardware and operates autonomously under variable environmental conditions. Extensive simulations using real-world weather, solar, and fire datasets demonstrate that EcoWild achieves up to 7.7× faster detection than static baselines while maintaining moderate battery energy levels across all scenarios. Notably, EcoWild avoids battery depletion in 125 diverse deployment locations, highlighting its robustness and sustainability. These results confirm that EcoWild is a practical and effective framework for enabling real-time wildfire monitoring in energy-constrained, infrastructure-limited environments.

## Figures and Tables

**Table 1:** State vector features with units and notation.

Feature (Unit)	Symbol
Temperature (°C)	*T*
Relative Humidity (%)	*H*
Wind Speed (km/h)	*W*
Hot-Dry-Windy Index	HDWI
Time of Day (normalized [0–1])	$t_{\mathrm{day}}$
Season (categorical)	$s_{\mathrm{season}}$
Harvested Energy from Solar Panels (Wh)	$E_{\mathrm{harvest}}$
Battery Energy (Wh)	$E_{\mathrm{battery}}$
Elapsed Time Since Last Image Capture (min)	$t_{\mathrm{lastCapture}}$
Previous Sampling Period (min)	$t_{\mathrm{prev}}$
Previous SD Result (binary: 1 = smoke, 0 = none)	$y_{\mathrm{prev}}$

**Table 2:** Smoke detection model settings for time vs. energy optimization.

Setting	Description	TPR	FPR
SD-time	Aggressive for fast detection	0.90	0.58
SD-energy	Conservative for low energy consumption	0.66	0.33

**Table 3:** Comparison of the detection algorithm components.

Algorithm	Fixed Sampling Period	DT	ML	RL
Fixed	✓	✗	✗	✗
DT-only	✓	✓	✗	✗
SD-time	✓	✗	✓ *	✗
SD-energy	✓	✗	✓ **	✗
DT-SD-time	✓	✓	✓ *	✗
DT-SD-energy	✓	✓	✓ **	✗
**EcoWild (Ours)**	✗	✓	✓	✓

* High TP/FP for faster detection. ** Low TP/FP for energy efficiency.

**Table 4:** Reward-related parameters used for training.

Parameter	$\alpha_{B}$	$R_{\min}$	β	*k*
**Value**	525,600	5000	0.9	100

## Data Availability

This study generated original simulation data (EcoWild results and processed energy traces), available upon request from the corresponding author. The fire image dataset (FigLib) is publicly available at https://www.hpwren.ucsd.edu/FIgLib/. Weather data were obtained from the Open-Meteo API (https://open-meteo.com), which is publicly accessible.
